# Research on Classification and Identification of Crack Faults in Steam Turbine Blades Based on Supervised Contrastive Learning

**DOI:** 10.3390/e26110956

**Published:** 2024-11-06

**Authors:** Qinglei Zhang, Laifeng Tang, Jiyun Qin, Jianguo Duan, Ying Zhou

**Affiliations:** China Institute of FTZ Supply Chain, Shanghai Maritime University, Shanghai 201306, China; qlzhang@shmtu.edu.cn (Q.Z.); 202230210067@stu.shmtu.edu.cn (L.T.); jyqin@shmtu.edu.cn (J.Q.); jgduan@shmtu.edu.cn (J.D.)

**Keywords:** supervised contrastive learning, channel attention mechanism, 1DCNN, fault diagnosis

## Abstract

Steam turbine blades may crack, break, or suffer other failures due to high temperatures, high pressures, and high-speed rotation, which seriously threatens the safety and reliability of the equipment. The signal characteristics of different fault types are slightly different, making it difficult to accurately classify the faults of rotating blades directly through vibration signals. This method combines a one-dimensional convolutional neural network (1DCNN) and a channel attention mechanism (CAM). 1DCNN can effectively extract local features of time series data, while CAM assigns different weights to each channel to highlight key features. To further enhance the efficacy of feature extraction and classification accuracy, a projection head is introduced in this paper to systematically map all sample features into a normalized space, thereby improving the model’s capacity to distinguish between distinct fault types. Finally, through the optimization of a supervised contrastive learning (SCL) strategy, the model can better capture the subtle differences between different fault types. Experimental results show that the proposed method has an accuracy of 99.61%, 97.48%, and 96.22% in the classification task of multiple crack fault types at three speeds, which is significantly better than Multilayer Perceptron (MLP), Residual Network (ResNet), Momentum Contrast (MoCo), and Transformer methods.

## 1. Introduction

As the most prominent component of the energy system, the production and optimal use of electrical energy is of vital importance, and large steam turbines serve as essential conversion devices in turbine power systems. Therefore, the reliability of large steam turbines is the main maintenance issue of power plants and an important continuous investment project [1]. During the operation of a steam turbine, a minor blade failure may result in a decrease in operational efficiency, while a significant failure could lead to a catastrophic incident, incurring substantial losses. It is essential that preventive measures are implemented to mitigate such risks and ensure the safe and efficient functioning of turbine systems.

Traditional manual detection relies on experience and subjective judgment, is easily affected by human factors, and has difficulty processing complex nonlinear data. In addition, studies have shown that the interaction between blades will aggravate crack propagation, and most defect-induced failures originate from fatigue crack propagation in stress concentration areas. When analyzing the classification of turbine blade crack failures, it is also necessary to consider the impact of the loading path on the classification results. The study by Y Zhang et al. [2] explored the interaction between the initial crack size, stress, and cracks in offshore pipelines and emphasized that the interaction between cracks will accelerate crack propagation and lead to larger cracks. This study has important guiding significance for the study of turbine blade cracks.

However, deep learning can adapt to new data patterns and process large-scale high-dimensional data through automatic feature extraction and training, outperforming traditional methods. Therefore, a fault diagnosis method based on deep learning shows great potential in improving the reliability and safety of rotating machinery [3,4,5]. Based on the in-depth analysis of the application value and practical significance of steam turbine blade faults, it is particularly important to optimize the fault classification algorithm and improve the accuracy of fault diagnosis.

Fault classification is a crucial link in the fault diagnosis process, and selecting appropriate classification algorithms is significant for the identification of fault features. In recent years, research on fault identification and classification algorithms has been significantly strengthened [6]. With the rapid development of artificial intelligence and deep learning technologies, more and more researchers have begun to apply these cutting-edge methods to the field of fault diagnosis. Various neural network-based classification algorithms, such as convolutional neural network (CNN) [7], long short-term memory network (LSTM) [8], and Transformer structure [9], have demonstrated excellent performance in fault classification, significantly improving accuracy and robustness. Particularly, the introduction of the attention mechanism enables the model to effectively capture key features, thereby further improving the effect of fault diagnosis. In addition, as an emerging unsupervised learning method, contrast learning (CL) improves the model’s fault identification ability under complex working conditions by optimizing the similarity and difference between samples.

In deep neural networks, CNNs are favored for their exceptional generalization capabilities and are widely applied across various fields. Originally proposed by LeCun [10], CNN was designed to process data with grid-like structures. Eren et al. [11] used CNN for fault diagnosis of gearbox bearings and gears, achieving a 6% improvement in classification accuracy. To overcome the limitations of manual feature extraction, an adaptive deep CNN was developed by Wang et al. [12] which automatically adjusts model parameters through Particle Swarm Optimization (PSO) to achieve feature learning. t-SNE was utilized for the visualization of hierarchical features, and this approach was applied to rolling bearing fault diagnosis. Dao et al. [13] proposed a fault diagnosis model based on Bayesian Optimization (BO) which combines CNN and LSTM to diagnose turbine faults. CNN extracts features, which are then used to train LSTM. The BO algorithm is used to optimize the hyperparameter selection of the model, solving the challenge of model parameter adjustment. Although CNN performs well, it encounters the issue of gradient vanishing or exploding during deep convolution, which limits the effectiveness of fault classification. Therefore, the one-dimensional convolutional neural network (1DCNN) was proposed to simplify the architecture of traditional CNNs. It is specifically designed for processing time series data or one-dimensional signals, utilizing convolutional layers for automatic feature extraction. This makes 1DCNN particularly effective for tasks such as classification and anomaly detection, especially in the field of health monitoring. Reference [14] proposed a rolling bearing fault diagnosis model based on adaptive modified complementary ensemble empirical mode decomposition (AMCEEMD) and 1DCNN. The selected intrinsic mode function (IMF) features were input into 1DCNN for fault classification. The results showed that the classification accuracy of the AMCEEMD-1DCNN method was better than other methods. In addition, Chen et al. [15] proposed a deep learning model 1DCNN-BiLSTM for detecting small local structural changes of reinforced concrete (RC) beams. They applied the Inception module structure in GoogLeNet to 1DCNN, automatically extracted spatiotemporal features from the signal, and accurately identified the location of local changes. The accuracy rate in the test set reached 98.8%, demonstrating excellent noise resistance and robustness to missing data.

In order to further enhance the feature extraction capability of complex signals, the attention mechanism enhances the representation capability of key features by weighting them in the channel dimension, thereby significantly improving the classification performance [16]. Specifically, Channel Attention Mechanism (CAM) focuses on the most informative channels in the feature maps, allowing the model to weigh the importance of different features more effectively, thus improving classification accuracy and enabling better feature representation. In addition, the attention mechanism can dynamically adjust the weight of each feature, allowing the model to focus more accurately on key information, thereby further improving classification accuracy [17]. Zhang et al. [18] proposed an electro-hydraulic steer-by-wire (EH-SBW) fault diagnosis method based on 1DCNN-LSTM, combining attention mechanism and transfer learning, using the scaled attention layer to amplify key features and reduce the impact of dual actuator coupling on the diagnosis results. Yao et al. [19] proposed a data-driven model that uses an Adam optimizer with separate weight decay and a phased learning rate scheduling strategy for training, which accurately reveals the aerodynamic performance of the blade tip and exhibits extremely high reliability. However, in industrial environments, due to safety considerations, it is common for machine owners not to continue operation when equipment fails, resulting in a significantly higher number of healthy samples compared to fault samples [20]. This data imbalance poses a serious limitation on the performance of deep learning models. Contrast learning, as an unsupervised learning method, has been shown to exhibit significant advantages in addressing data imbalance issues, thus providing a new research direction for further enhancing model performance.

Contrastive learning, as a self-supervised learning method, has attracted widespread attention in recent years. This method generates positive and negative sample pairs through data augmentation techniques and trains the network to enhance the distinction between negative samples while reducing the distance between positive samples, thereby being able to extract discriminative features from unlabeled data [21]. Supervised Contrastive Learning (SCL), on the other hand, leverages labeled data to learn feature representations by maximizing the agreement between similar samples and minimizing it between dissimilar ones. This approach enhances the model’s ability to distinguish between different classes, improving generalization performance in classification tasks. He et al. [22] proposed Momentum Contrast (MoCo) in 2020. Self-supervised learning methods represented by contrastive learning have been widely studied in the image field and have shown significant advantages over supervised learning methods. However, to date, the application of contrastive learning methods in the field of fault diagnosis is still relatively rare. Ding et al. [23] developed MoCo as a detection method for early rolling bearing faults. Liu et al. [24] proposed a new method based on meta-analogy momentum contrast learning (MA-MoCo). By improving the MoCo method and using the training idea of meta-learning, it was applied to the fault diagnosis of wind turbine transmission systems. An et al. [25] proposed a domain adaptive network (DACL) based on contrastive learning, aiming to achieve cross-condition fault diagnosis and reduce the classification error of samples near or on the boundaries of various types to improve the diagnosis accuracy. In addition, Zhang et al. [26] also applied MoCo to the multi-scale convolutional bearing fault diagnosis structure. In the case of limited labeled data, the accuracy of fault diagnosis was significantly improved by suppressing irrelevant information and enhancing the contribution of important features.

**Figure 1:**
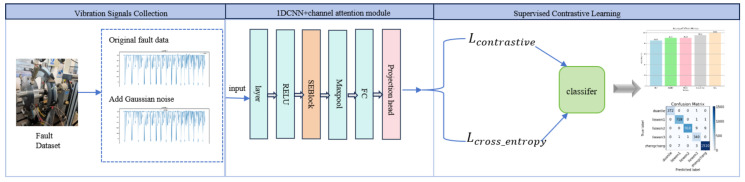
System framework diagram (The figure illustrates a fault diagnosis framework based on vibration signal data. The experiment collects vibration signals of blade crack faults, constructs a fault dataset, and adds Gaussian noise. The 1D CNN is combined with CAM to extract fault features. A projection head is introduced to map all sample features into a normalized space, thereby enhancing the model’s ability to distinguish between different fault types. Additionally, contrast loss and cross-entropy loss are calculated through supervised contrast learning to complete the fault classification.).

In summary, in order to effectively solve the problem of imbalanced fault data types, this paper proposes a contrastive learning method that combines one-dimensional CNN with an attention mechanism. The effectiveness of the proposed method is verified using blade crack fault data collected in a laboratory environment. The framework is shown in Figure 1.

The main contributions of this paper are as follows:

1. The blade crack fault data collected in the laboratory were processed to generate fault datasets at three different speeds. The fault samples were augmented using data enhancement techniques and other methods to address the data imbalance issue, thereby enhancing the model’s capacity to classify and identify faults effectively.
2. We introduce the Channel Attention Mechanism (CAM), assigning different weights to each channel, highlighting important features, and improving the overall performance of the model;
3. By introducing contrastive learning and combining 1DCNN and CAM with the original cross entropy loss function, the model can better capture the subtle differences between different types of faults, further improving the performance of the model.

The rest of this article is organized as follows:

Section 2 provides a detailed introduction to the research methodology. Section 3 demonstrates the superiority of the proposed method through experimental comparisons. Finally, Section 4 presents the conclusions and discusses future development prospects.

## 2. Method

This paper primarily integrates 1DCNN, CAM, and SCL method to process vibration signals for the classification of turbine blade crack faults. Specifically, the 1DCNN is utilized to capture local features within the vibration signals, while CAM enhances attention to crucial features. By combining these approaches with SCL, we improve the model’s classification capability for complex fault modes. The integration of CAM with 1DCNN has been effectively applied in health monitoring, significantly enhancing the model’s ability to focus on key features in time-series data. This integration not only improves classification accuracy but also enhances feature representation, particularly in anomaly detection tasks. Prior studies have demonstrated the effectiveness of CAM in improving model performance in health monitoring applications.

This paper proposes a novel methodology that combines SCL with CAM and 1DCNN. This combination effectively addresses challenges associated with data imbalance, as SCL enhances the model’s ability to differentiate between categories by maximizing similarity among similar samples while minimizing it among dissimilar ones. The proposed method not only boosts classification performance in fault diagnosis but also provides a robust solution for tackling data imbalance, a common challenge in health monitoring. Additionally, data augmentation techniques, such as the addition of Gaussian noise, are employed to expand the dataset, further enhancing the model’s robustness and generalization capabilities. The following sections provide a detailed description and parameter settings for each method.

### 2.1. DCNN

CNN is a deep learning model particularly well-suited for processing time series and spatial data, efficiently extracting local features from the input. In one-dimensional signal processing tasks, the core of the 1D CNN lies in the convolution operation, which performs sliding window scanning on the input vibration signal. By utilizing local receptive fields and parameter sharing, it effectively captures local features of the input data. The mathematical expression for the convolution operation is as follows:

(1) $$Y_{i}=f\left(\sum_{m=0}^{M-1}W_{m}\cdot X_{i+m}+B\right)$$

Y[i] represents the output feature at position i; M is the number of weights in the convolution kernel; $W_{m}$ is the *m*th weight in the convolution kernel; $X_{i+m}$ is the value of the input signal at position i+m; B is the bias term. Through the convolution operation, the model can effectively extract local features, and by superimposing multiple layers of convolution in the entire network, 1DCNN can capture deeper abstract features.

The first convolutional layer in this paper processes the raw vibration data of the input signal, featuring 1 input channel, 64 output channels, 3 convolution kernels, a padding of 1, and a ReLU activation function. The second convolutional layer consists of 64 input channels, 128 output channels, and also utilizes 3 convolution kernels with a ReLU activation function. This layer further extracts higher-level features. After each convolutional layer, a max pooling operation is applied, which reduces the dimensionality of the feature map by selecting the maximum value within a local area. This process decreases computational complexity while preserving the essential features.

Output size calculation after convolution operation:

(2) $$W_{out}=\frac{W_{in}+2P-F}{S}+1$$

This formula is used to calculate the size of the output feature map after the convolution operation. $W_{out}$ represents the width of the output feature map; $W_{in}$ represents the width of the input feature map; P represents the padding added to the edge of the input feature map, which is used to control the spatial size of the output; F is the size of the convolution kernel; S is the stride, which determines the distance the convolution kernel moves each time. The CNN back-propagation algorithm uses gradient information to optimize the network weights, minimize the loss function on the training data, and reduce the difference between the predicted value and the true value. The back-propagation weight is updated as follows:

(3) $$W_{new}^{l}=W_{old}^{l}-\eta \left(\frac{\partial C}{\partial W_{old}^{l}}+\lambda \cdot W_{old}^{l}\right)$$

$W_{new}^{l}$ represents the weight matrix of the lth layer after update; $W_{old}^{l}$ represents the weight matrix of the lth layer before update; η represents the learning rate; $\partial C/\partial W_{old}^{l}$ is the gradient of the loss function C with respect to the weight matrix $W_{old}^{l}$, indicating the direction and magnitude of weight adjustment required to reduce the loss; $\lambda \cdot W_{old}^{l}$ represents the regularization term used to prevent overfitting; λ is the regularization parameter, whose purpose is to control the intensity of this penalty.

### 2.2. CAM

The attention mechanism is a dynamic weighting process that optimizes the network’s feature acquisition by assigning weights to the extracted feature maps, thereby enhancing recognition accuracy. CAM is a technique designed to improve the performance of CNN models. It primarily applies weighting to the channel dimension of feature maps to emphasize key features, thus increasing the model’s expressiveness and classification performance. The fundamental idea of CAM is to enable the model to automatically learn and identify which feature channels are more relevant to the current task, assigning these channels higher weights [27,28].

CAM includes the following steps:

(4) $$z_{c}=GlobalAvgPool\left(X_{c}\right)=\frac{1}{H\times W}\sum_{i=1}^{H}\sum_{j=1}^{W}X_{c}\left(i,j\right)$$

First, perform a global average pooling operation on the input feature map to generate a channel-level global feature vector, where $z_{c}$ is the global feature of channel c, $X_{C}\;(i,j)$ is the value of the input feature map X at channel c and spatial position (i, j), and H and W are the height and width of the feature map, respectively.

(5) $$\omega_{c}=\sigma \left(FC\left(z_{c}\right)\right)$$

$\omega_{c}$ is the attention weight of channel c, σ is the activation function that limits the weight range to between 0 and 1, and FC is the fully connected layer.

(6) $${\hat{X}}_{C}=\omega_{c}\cdot X_{c}$$

The channel attention weight $\omega_{c}$ is multiplied by the corresponding channel of the input feature map to complete the feature recalibration. ${\hat{X}}_{C}$ is the output feature of channel c after recalibration. The introduction of CAM enables the model to capture meaningful features more effectively when processing data with high dimensions and complex structures, thereby improving the overall performance of the model.

### 2.3. SCL

SCL is a deep learning method that applies contrastive learning to supervised tasks. It leverages the benefits of both supervised and contrastive learning to enhance the classification performance of the model. This approach is especially effective for tasks involving labeled data, such as image classification and text classification [29,30]. The loss function of contrastive learning is expressed as follows:

(7) $$L_{contrastive}=\sum_{i\in I}\frac{1}{\left|P\left(i\right)\right|}\sum_{p\in P\left(i\right)}\left[-log\frac{\exp\left(\frac{sim\left(z_{i},z_{p}\right)}{\tau }\right)}{\sum_{a\in A\left(i\right)}\exp\left(\frac{sim\left(z_{i},z_{a}\right)}{\tau }\right)}\right]$$

I is a set of sample indices; P(i) is a set of positive samples belonging to the same category as sample i; A(i) is a set of all samples related to sample i (including positive and negative samples); $z_{i}$ is a feature representation of sample i; $sim(z_{i},z_{p})$ represents the similarity between sample i and positive sample p; τ is a temperature parameter; $exp(sim(z_{i},z_{p})/\tau )$ converts the similarity value into a probability value through an exponential function to calculate the contrast loss.

Cross-entropy loss is integrated into contrastive learning, directly utilizing category labels to optimize the model. The optimization process involves calculating the difference between the category probability distribution predicted by the model and the true label distribution, enabling the model to more accurately predict the category of each sample.

(8) $$L_{cross_{entropy}}=-\sum_{i=1}^{N}\sum_{c=1}^{C}y_{ic}\log\left({\hat{y}}_{ic}\right)$$

N represents the number of samples, indicating the total number of samples in the dataset, while C denotes the number of categories, reflecting the total number of categories in the classification task. The variable $y_{ic}$ serves as an indicator for the true label; specifically, if the true category of sample i is c, then $y_{ic}=1$; otherwise, $y_{ic}=0$. The variable $y_{ic}$ represents the predicted probability of sample i belonging to category c, which is typically obtained from the model’s output layer.

This paper also introduces the projection head, a network component designed to embed data into the feature space. Its primary function is to map the original features into a new space, facilitating improved contrastive learning and classification tasks [31].

In this paper, we propose a model based on 1DCNN and CAM. The model structure includes two convolutional layers with 64 and 128 filters, respectively, with a kernel size of 3, and a ReLU activation function and SE module are added after each convolutional layer. A 2 × 2 max pooling layer is applied after each convolutional layer to reduce the size of the feature map. Regarding the fully connected layers, the first layer maps the feature dimension from 128 times 45 to 128 dimensions according to the specific feature length of the input data. Subsequently, we introduce a projection head, and two fully connected layers map the features from 128 dimensions to 256 dimensions and then to 512 dimensions, respectively. Finally, a fully connected layer maps the features to the corresponding number of categories.

We use the Adam optimizer to train the model with a learning rate set to 0.001. In addition, the cross entropy loss function is used for loss calculation of classification tasks, combined with the supervised contrast loss function to enhance the ability of feature representation. The specific training process includes forward propagation of each batch of input data, and backpropagation after calculating the loss to update the model parameters. In the experiment, the calculations include training loss, test loss, training accuracy, F1 score, precision, and recall. These indicators help evaluate the performance of the model in the time series classification task and provide a basis for subsequent optimization.

## 3. Data Augmentation

Data Augmentation is one type of data augmentation, which is currently one of the most advanced data augmentation methods. A primary objective is to expand the scale of the training dataset, enhance the model’s generalization ability, and reduce the risk of overfitting. In the field of image processing, data augmentation has been proven effective; techniques such as random cropping, resizing, and color distortion can significantly improve model performance [32]. However, many methods used for augmenting two-dimensional image data are not entirely applicable to one-dimensional vibration signals, necessitating careful selection of appropriate augmentation strategies when handling different types of data.

Researchers have explored some data augmentation methods suitable for one-dimensional signals [33], such as Gaussian noise, amplitude scaling, X-Y flipping, generative adversarial networks, DC offset, etc. Gaussian noise, as a common natural noise model, plays an important role in enhancing the robustness of the model. By adding Gaussian noise to the input data or features during training, the smoothness of the data can be broken, preventing the model from overfitting, thereby forcing the model to learn more generalized feature representations. In addition, Gaussian noise can also be used to generate diverse training samples, especially when data is scarce, to effectively expand the size of the data set and improve the performance of the model. For applications that require capturing subtle differences, such as fault diagnosis, the addition of Gaussian noise can enhance the model’s ability to effectively manage small perturbations in the input data.

**Figure 2:**
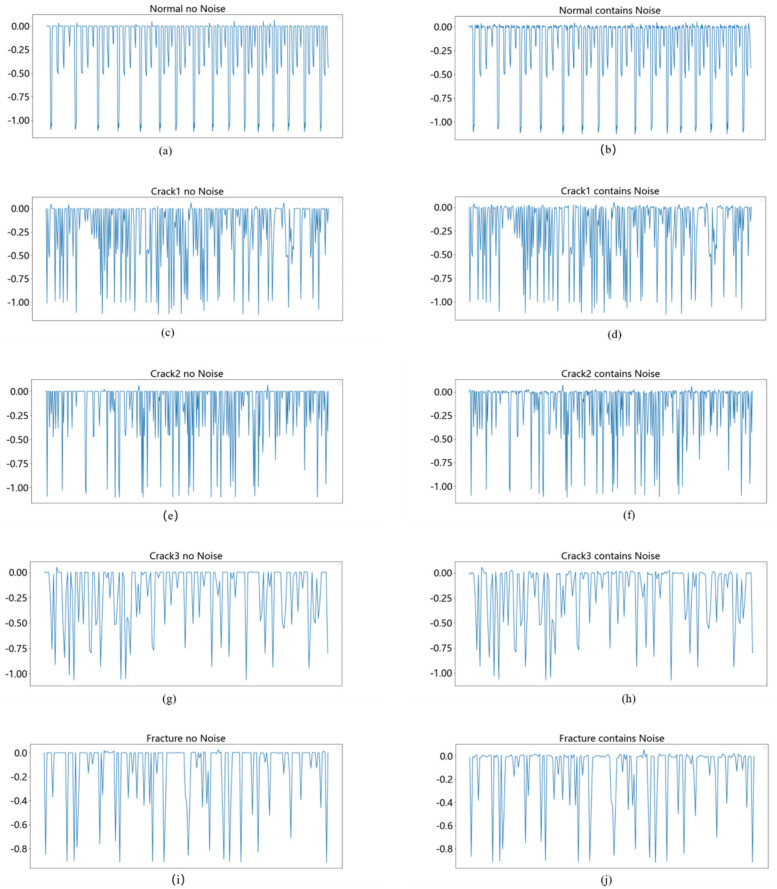
Vibration signals before and after adding Gaussian noise. ((**a**,**c**,**e**,**g**,**i**) represent the “normal” without adding Gaussian noise, the fault signal of “crack1”, the fault signal of “crack2”, the fault signal of “crack3”, and the fault signal of “fracture”, respectively; (**b**,**d**,**f**,**h**,**j**) represent the above five signals after adding Gaussian noise, respectively.).

As shown in Figure 2, this paper adopts a data enhancement method of adding Gaussian noise. Given a vibration signal $X=[x_{1},x_{2},\ldots ,x_{l}]$, noise $N(0,\sigma_{n}^{2})$ with Gaussian distribution is added to the original signal. The length of the noise is equal to the original signal, $\hat{X}=X+\epsilon ,\;\epsilon \sim N(0,\sigma_{n}^{2})$, X is the original signal; ϵ is the noise, which obeys the Gaussian distribution $N(0,\sigma_{n}^{2})$ with mean 0 and variance $\sigma_{n}^{2}$. The standard deviation $\sigma_{n}$ of the noise determines the intensity of the noise, and $\hat{X}$ is the signal after adding noise.

## 4. Experiment

### 4.1. Data Collection and Experimental Details

**Figure 3:**
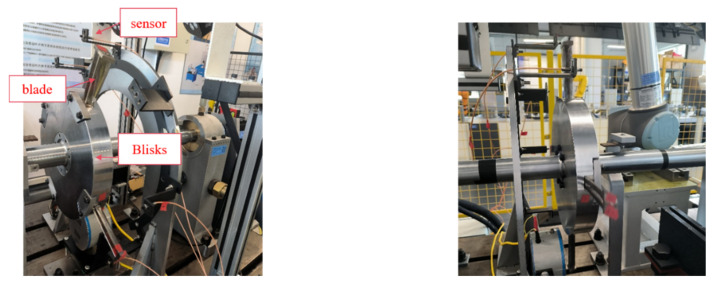
Dynamic test bench of rotor system with integral shroud blade.

To evaluate the effectiveness of the proposed method, experiments were carried out on a dynamic test bench of a rotor system with an integral shroud blade in the laboratory. The data set test bench is shown in Figure 3. Five groups of blade failure data at three speeds of 1400 r/min, 1800 r/min, and 2200 r/min were tested, and the blade parameters are shown in Table 1. Two blades form a group, and the interval between each group of blades is 90°. The blades are fixed to the blade disk with cast iron block baffles and screws, and five eddy current displacement sensors are installed to collect blade vibration displacement signals, with a sampling frequency of 50 KHz.

The vibration displacement data of five sets of blades collected at three different speeds were filtered to exclude signals that were not captured by the sensors (signals with a value of 0). After adding Gaussian noise, wavelet transformation was applied to extract fault features.

### 4.2. Performance Comparison Test

**Figure 4:**
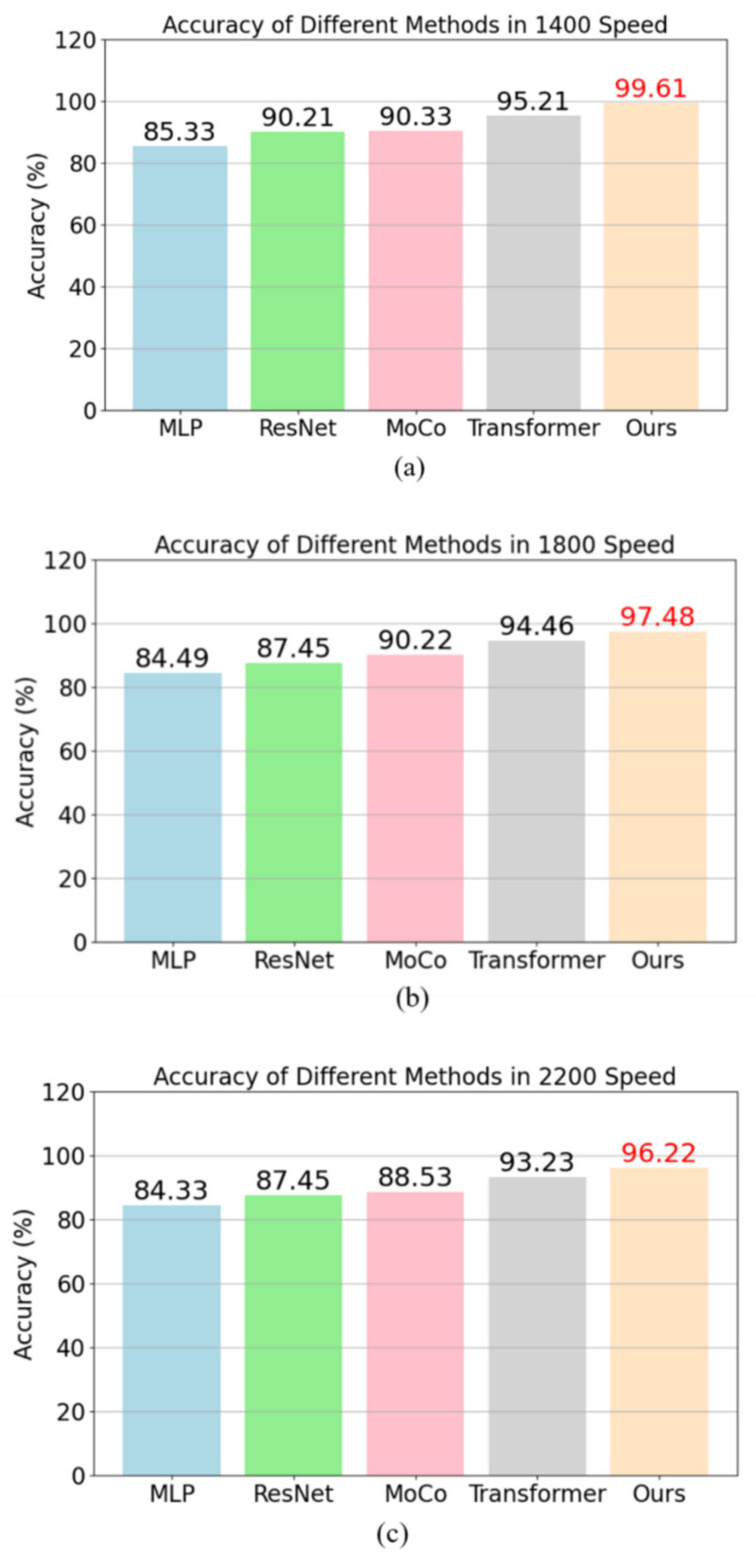
(**a**–**c**) represent the accuracy of MLP, ResNet, MoCo, Transformer, and our proposed method at 1400 r/min, 1800 r/min, and 2200 r/min, respectively.

To evaluate the fault classification capability of this method, comparative experiments were conducted with several other common supervised learning techniques. The methods compared include Multilayer Perceptron (MLP) [34], Residual Network (ResNet) [35], MoCo, and Transformer. All models followed the same training and testing process, in which 80% of the fault dataset was used for model training, while the remaining data served as the test set. The results of the comparative experiments are presented Figure 4. In addition to accuracy, precision, recall, and F1 score are also critical indicators for assessing model performance.

The definition of $F_{1}$-score is as follows:

(9) $$Precision=\frac{TP}{TP+FP}$$

(10) $$Recall=\frac{TP}{TP+FN}$$

(11) $$F_{1}=2\frac{Precision\cdot Recall}{Precision+Recall}$$

TP, FP, and FN represent true positive, false positive, and false negative, respectively. For multi-class classification tasks, the aforementioned formula can be applied to each category, and the average value is then calculated. The experimental results are presented in Table 2, Table 3 and Table 4.

The results show that the performance of MLP is relatively weak, with all indicators at around 85% at three speeds; ResNet has significant improvements in all indicators, reaching around 90%; the performance of the MoCo method is slightly better than ResNet, especially in F1 score and accuracy, indicating that the complex model structure is more helpful in improving the accuracy and robustness of fault classification; the results of Transformer are relatively high, indicating that the introduction of attention mechanism can further improve the model performance, but it is still lower than the proposed method. As a one-dimensional convolutional neural network, 1DCNN can efficiently extract local features from time series signals. Compared to models such as MLP and ResNet, 1DCNN is more suitable for handling one-dimensional data like vibration signals, as it effectively captures crucial features within fault signals. By incorporating the attention mechanism and supervised contrastive learning, the proposed method enhances classification accuracy and F1 scores. It performs exceptionally well at all three speeds, particularly surpassing ResNet and MoCo in F1 scores and precision. This indicates that the method offers greater accuracy and robustness in distinguishing subtle differences in fault diagnosis tasks under complex operating conditions.

From the table, it is evident that the evaluation metrics for each fault classification are above 96%, clearly indicating that the method demonstrates excellent diagnostic performance on the fault dataset and validating the effectiveness of the proposed approach.

**Figure 5:**
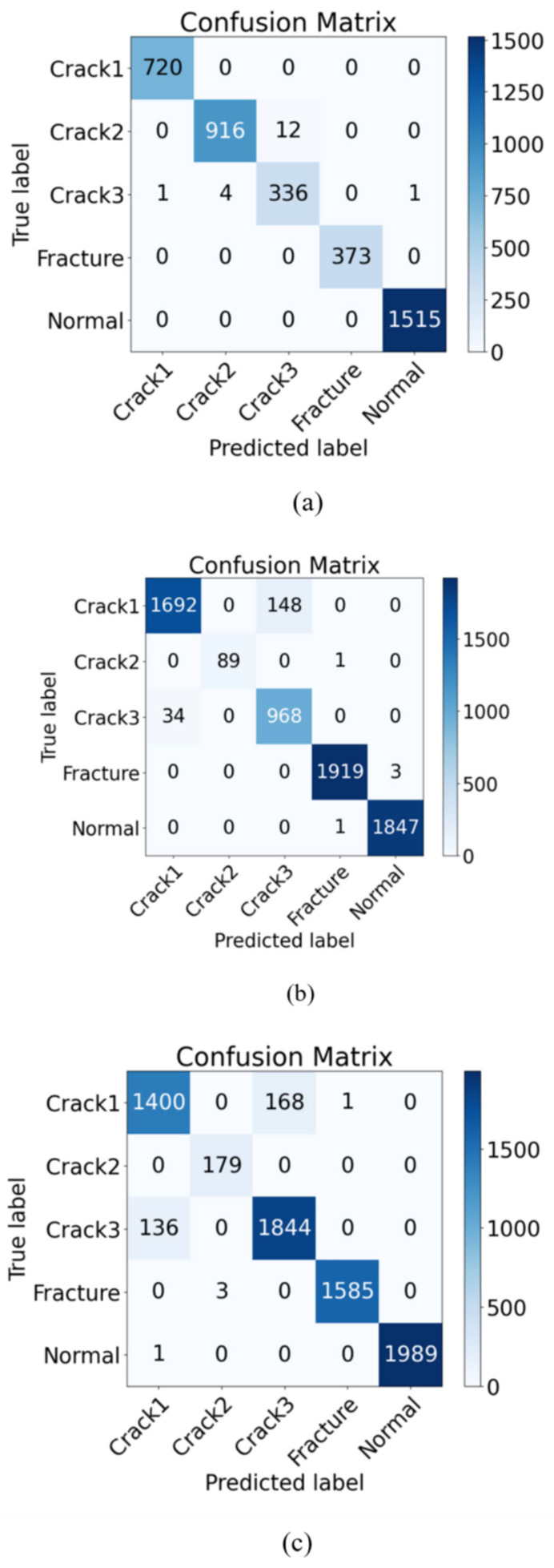
(**a**–**c**) represent the confusion matrix results at 1400 r/min, 1800 r/min, and 2200 r/min. The values on the diagonal represent the number of samples predicted correctly, and the values on the off-diagonal represent the number of samples predicted incorrectly.

Figure 5 presents the confusion matrix for the proposed method, which was trained on data from three different speeds. The results indicate that the classification performance for the “fracture” and “normal” categories demonstrate a high level of stability, with nearly no misclassifications. This suggests that the model excels at distinguishing between obvious faults and normal states. However, there are some misclassifications among“crack1”, “crack2”, and “crack3”, likely due to the relatively similar features of these three categories. Overall, the classification performance remains strong.

### 4.3. Ablation Experiment

In order to evaluate the impact of each module in the proposed method on the diagnostic performance, this section uses three speed datasets for ablation experiments. Specifically, the accuracies without considering CAM and SCL are 95.33%, 95.12%, and 93.07%, respectively. When CAM is excluded and SCL is used, the classification accuracies rise to 97.11%, 95.95%, and 94.73%, respectively. In contrast, when CAM is introduced and SCL is removed, the accuracies are 98.31%, 96.33%, and 95.85%, respectively. Finally, when CAM and SCL are used simultaneously, the classification accuracies reach 99.61%, 97.48%, and 96.22%. The ablation experiment results of the three speed datasets are shown in Table 5, Table 6 and Table 7. Obviously, the removal of these two modules leads to a decrease in classification performance, which strongly demonstrates the effectiveness of the model’s classification ability.

### 4.4. Noise Ablation Experiment

In practical applications, data is often interfered with by various noises. The experimental results are shown in Table 8. When Gaussian noise is not added, the classification accuracy is lower than that after adding Gaussian noise. This shows that the introduction of noise can be regarded as a means of data enhancement, which increases the diversity of training data and enhances the generalization ability of the model. This method enables the model to effectively capture underlying patterns in the data while mitigating the risk of overfitting, allowing it to maintain a high accuracy rate even in complex practical environments and demonstrating strong robustness.

### 4.5. Visualization Analysis

**Figure 6:**
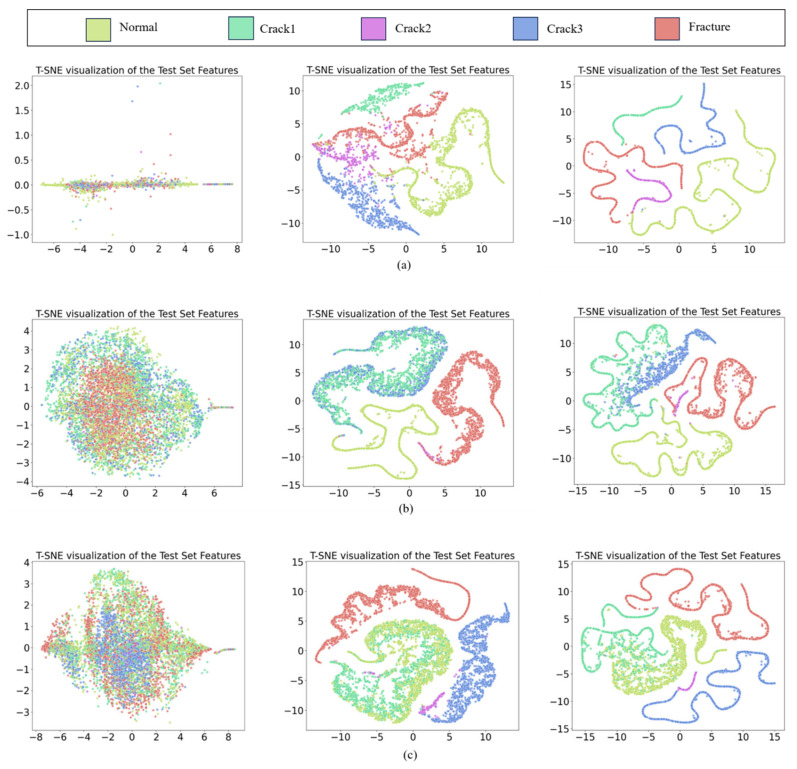
t-SNE feature visualization: (**a**) 1400 r/min, (**b**) 1800 r/min, (**c**) 2200 r/min.

To further demonstrate the superiority of the proposed method, t-SNE is used to reduce the dimension of the extracted high-dimensional features and visualize them. First, the signal features at three speeds are visualized, and then the same signal features extracted by the training model are visualized. The feature visualization is shown in Figure 6. Each color represents a different fault category. The original signal is difficult to distinguish. After training, most of the features of the samples are clustered together by category. This result shows that the model can obtain good learning ability through training. The third sub-figure shows that the features are clearly clustered by category, indicating that the fine-tuning process can further improve the model’s feature extraction ability. In summary, this method can gradually develop feature extraction capabilities through training and fine-tuning.

## 5. Conclusions

A blade crack fault classification method combining 1DCNN, CAM and SCL is proposed to solve the problem of insufficient labeled data in industrial scenarios. Firstly, Gaussian noise is added to enhance the data of the collected vibration signal, and the non-stationary features related to the fault are extracted by wavelet transform. The wavelet coefficient matrix is input into 1DCNN, and combined with its powerful feature extraction ability, signal classification is achieved. By introducing CAM, the model adaptively adjusts the channel weights and enhances the expression of key fault features. In addition, a supervised contrastive learning strategy is adopted to map samples to the normed space and construct pairs of similar and heterogeneous samples to further improve the model’s discrimination ability. Experimental results show that under the condition of limited labeled data, this method improves the classification accuracy compared with traditional deep learning methods. Visual experiments verify the effectiveness and interpretability of the model and demonstrate its robustness and application potential under complex working conditions. However, this method still relies on labeled data for training, making it challenging to identify faults that are not present in the training set. Therefore, future research should explore the application of contrastive learning in areas such as small sample learning, domain adaptation, and unknown fault identification.

## Figures and Tables

**Table 1:** Blade parameters.

Blade Failure Type	Leaf Label
Normal leaves	normal
Crack depth 5.8 × 1 × 0.5 mm (length, width, depth)	crack1
Crack depth 10.8 × 1 × 0.5 mm (length, width, depth)	crack2
Crack depth 5.8 × 1 × 0.8 mm (length, width, depth)	crack3
Broken blade	fracture

**Table 2:** Comparison results of performance indicators of different fault classification methods at 1400 speed.

Methods	Accuracy	F1	Precision	Recall
MLP	85.33	84.19	84.19	84.32
ResNet	90.21	89.98	89.98	90.47
MoCo	90.33	90.47	90.65	90.53
Transformer	95.21	95.21	95.21	95.21
Proposed method	99.61	99.61	99.61	99.61

**Table 3:** Comparison results of performance indicators of different fault classification methods at 1800 speed.

Methods	Accuracy	F1	Precision	Recall
MLP	84.49	83.92	84.76	84.19
ResNet	87.45	87.81	88.62	87.21
MoCo	90.22	90.22	90.22	90.22
Transformer	94.46	94.21	94.46	94.21
Proposed method	97.48	97.50	97.52	97.48

**Table 4:** Comparison results of performance indicators of different fault classification methods at 2200 speed.

Methods	Accuracy	F1	Precision	Recall
MLP	84.33	84.19	84.44	84.33
ResNet	87.45	87.81	86.81	88.81
MoCo	88.53	88.47	89.6	88.53
Transformer	93.23	93.37	94.48	93.23
Proposed method	96.22	96.22	96.22	96.22

**Table 5:** Ablation test results at 1400 r/min.

	Accuracy	F1	Precision	Recall
14001DCNN	95.33	96.39	95.93	96.86
14001DCNN + SCL	97.11	97.31	97.23	97.41
14001DCNN + CAM	98.31	98.22	98.21	98.23
14001DCNN + SCL + CAM	99.61	99.61	99.61	99.61

**Table 6:** Ablation test results at 1800 r/min.

	Accuracy	F1	Precision	Recall
18001DCNN	95.12	95.17	95.22	95.13
18001DCNN + SCL	95.95	95.85	95.73	95.98
18001DCNN + CAM	96.33	96.54	96.32	96.77
18001DCNN + SCL + CAM	97.48	97.50	97.52	97.48

**Table 7:** Ablation test results at 2200 r/min.

	Accuracy	F1	Precision	Recall
22001DCNN	93.07	93.17	93.23	93.11
22001DCNN + SCL	94.73	95.21	94.53	95.89
22001DCNN + CAM	95.85	95.85	95.85	95.85
22001DCNN + SCL + CAM	96.22	96.22	96.22	96.22

**Table 8:** Noise ablation results.

	Accuracy	F1	Precision	Recall
1400 without noise	95.67	95.60	95.58	95.62
1400 containing noise	99.61	99.61	99.61	99.61
1800 without noise	93.59	93.54	93.47	93.61
1800 containing noise	97.48	97.50	97.52	97.48
2200 without noise	92.43	92.45	92.39	92.41
2200 containing noise	96.22	96.22	96.22	96.22

## Data Availability

The data presented in this study are available from the corresponding author upon request. (Due to laboratory policy or confidentiality agreements, we do not provide raw data. We have fully described the experimental design, analysis, results, and data processing. If you have any questions, we will try our best to provide more detailed explanations).
